# IoT-Based COVID-19 Diagnosing and Monitoring Systems: A Survey

**DOI:** 10.1109/ACCESS.2022.3197164

**Published:** 2022-08-08

**Authors:** Nasreen Anjum, Mohammad Alibakhshikenari, Junaid Rashid, Fouzia Jabeen, Amna Asif, Ehab Mahmoud Mohamed, Francisco Falcone

**Affiliations:** Department of Cyber and Technical Computing, School of Computing and EngineeringUniversity of Gloucestershire, Park Campus, Cheltenham 2376 Gloucestershire GL50 2RH U.K.; Department of Signal Theory and CommunicationsUniversidad Carlos III de Madrid, 28911 Leganés 16726 Madrid Spain; Department of Computer and EngineeringKongju National University 65361 Cheonan 31080 South Korea; Department of Computer ScienceShaheed Benazir Bhutto Women University Peshawar Peshawar 00384 Pakistan; Department of Electrical EngineeringCollege of Engineering in Wadi AddawasirPrince Sattam Bin Abdulaziz University 204568 Wadi Addawasir 11991 Saudi Arabia; Department of Electrical EngineeringAswan University 435387 Aswan 81542 Egypt; Department of Electric, Electronic and Communication Engineering and the Institute of Smart CitiesPublic University of Navarre 16756 31006 Pamplona Spain; Tecnológico de MonterreySchool of Engineering and Sciences 294008 Monterrey 64849 Mexico

**Keywords:** COVID-19 pandemic, coronavirus, machine learning algorithms, artificial intelligence (AI), Internet of Things (IoTs)

## Abstract

To date, the novel Coronavirus (SARS-CoV-2) has infected millions and has caused the deaths of thousands of people around the world. At the moment, five antibodies, two from China, two from the U.S., and one from the UK, have already been widely utilized and numerous vaccines are under the trail process. In order to reach herd immunity, around 70% of the population would need to be inoculated. It may take several years to hinder the spread of SARS-CoV-2. Governments and concerned authorities have taken stringent measurements such as enforcing partial, complete, or smart lockdowns, building temporary medical facilities, advocating social distancing, and mandating masks in public as well as setting up awareness campaigns. Furthermore, there have been massive efforts in various research areas and a wide variety of tools, technologies and techniques have been explored and developed to combat the war against this pandemic. Interestingly, machine learning (ML) algorithms and internet of Things (IoTs) technology are the pioneers in this race. Up till now, several real-time and intelligent IoT-based COVID-19 diagnosing, and monitoring systems have been proposed to tackle the pandemic. In this article we have analyzed a wide range of IoTs technologies which can be used in diagnosing and monitoring the infected individuals and hotspot areas. Furthermore, we identify the challenges and also provide our vision about the future research on COVID-19.

## I. Introduction

First reported by officials in Wuhan, China, in December 2019, the “SARS-CoV-2” infection had quickly spread by late January 2020 to every region of China and numerous different nations [1], [2], [3]. As of 21st January 2021, 213 countries and territories have registered COVID-19 cases. On January 30, 2020, the World Health Organization (WHO) declared that the outbreak constitutes a Public Health Emergency of International Concern (PHEIC); and on 11th March 2019, the WHO declared the COVID-19 outbreak a “global pandemic” [4], [5]. By April 6, 2020, more than 1,200,000 individuals had been infected by the disease and, what is more, the number of deaths due to the Coronavirus exceeded 70,000 in over 213 nations. As of 3rd August 2022, in-spite of thorough worldwide regulation and isolated endeavors, the cases of COVID-19 keep on ascending globally, with 575,887,049 confirmed cases research facility affirmed cases and more than 6,398,412 deaths [6].

Due to this worldwide flare-up, more than 200 nations worldwide had initiated either a full lockdown or remote area lockdowns, affecting the lives of billions of people, directly and indirectly. It has also had significantly impacted the global economy and has been more extreme than the 2008 global financial crises (GFC)] [7]. The stock exchanges have been imploded by half or more, credit markets have been frozen, and a significant number of bankruptcies followed [8]. However, even though a few nations have forced “smart lockdowns” in certain areas with potential COVID-19 cases, there has still been a flood of new COVID cases and an ascent in deaths [9], [10], worldwide due to the emergence of new COVID-19 stains in different parts of the world such as UK [11], South Africa [12], Brazil [13], Japan [14], USA [15] and India (Indian variant) [16].

Currently, five vaccines, two from China, two from the U.S., and one from the UK, have already been widely used for inoculation across the world, according to the WHO [17]. The first batch of the COVID-19 vaccine has already been distributed and in order to reach herd immunity, around 70 percent of the population would need to be vaccinated which might take upto a few years. Since developing world does not have access to vaccines, coronavirus will still ravage many parts of the world. Furthermore, according to the White House briefing [18], these vaccines are less effective against the new COVID-19 strains.

Although boosters are being offered to the general pubic, yet they are catching the virus. Such an alarming situation would mean eradicating the virus may be impossible as new outbreaks emerge around the world and the potential for more contagious or virulent variants of SARS-CoV-2 becomes all the more dangerous. For instance as of 3rd August 2022 research scientists have discovered number of recombinant COVID-19 variants (hybrid variant of the Delta and Omricon Strains) named as XD, XE and XF in China, UK, and Europe [19]. As per the initial assessment of the research community, one of the recombinant variants is ten times more transmissible than its parent Omicron variant [20]. While vaccinations and boosters in combination with lockdown measures far and wide are forced to prevent new infections, yet countless efforts are required to prevent and end the COVID-19 pandemic.

In current conditions, intelligent, automated, and real-time systems which can diagnose the Corona infected individuals and monitor those who are already infected will give WHO and governments around the globe a useful tool to deal with the pandemic. Moreover, intelligent tools and technologies that help in monitoring and diagnosing the COVID-19 is the need of today. Hence, the investigations of the corona virus and diseases it caused, its propagation and mutation development pattern, fast and efficient monitoring and diagnosing systems, have become the front line research points right now and have received a generous amount of consideration and attention from data scientists and researchers from all around the globe. This paper aims at conducting an extensive survey of the monitoring and diagnosing systems proposed to prevent the infections caused by the Coronavirus in communities.

Internet of Thing (IoT) technology alongside its wearable sensor nodes and vision based technology (cameras) have effectively been utilized in monitoring and diagnosing constant illnesses, for example, patients experiencing Parkinson [22] and Alzheimer’s [23] disease, monitoring the blood glucose levels of diabetic patients [24], Respiratory rate [25], blood pressure [26], [27], [28], [29], [30], and detecting cardiovascular failure [31].^1^ Based on the advantages and the suitability of IoTs in healthcare systems, we have explored several research efforts towards developing IoT based COVID-19 monitoring and diagnosis healthcare systems. Therefore, in this survey article, we have explored several research efforts towards developing IoT based COVID-19 monitoring and diagnosing healthcare systems.^1^For more information on the applications of IoTs in healthcare systems the intrigued reader may refer to [32].

### A. Comparison to Other COVID-19 Survey Articles

Since the COVID-19 outbreak (Dec-2019), a few published survey papers took a comprehensive review at the COVID-19 pandemic from various perspectives. For example, the authors in [33] and [34] have distinguished potential uses of Artificial Intelligence (AI) tools and technologies that can be utilized in COVID-19 immunization planning and drug discovery. The authors in [35] introduced a broad survey of cutting edge AI devices in forecasting and finding numerous viral illnesses. Authors recommended that artificial intelligence technology can be use to identify the protein structure of SARSCoV-2 and distinguish existing medicines that might helps to cure the infection. The authors in [36], discussed briefly uses of deep learning tools in diagnosing the COVID-19 infection. In [37], the authors provide a brief overview of the current status of IoT applications related to the Coronavirus, and suggest and identify some potential research areas to further investigate the outbreak. The authors in [38] presented a comprehensive study on open source data sets and their applications in mitigating the COVID-19 transmission.

In contrast, to help the research community, to have an overall comprehension of the continuous exploration and potential research areas in COVID-19, our major contributions in this article are as follow:

### B. Contributions

a) We provide an extensive review of the COVID-19 literature since the start of the pandemic. We have captured the most recent and highly cited research articles on IoT technologies used to develop COVID-19 early diagnosing and monitoring systems.
b) Unlike the previous studies [33], [35], [36], which have only emphasized on general aspects of COVID-19 related challenges in ML algorithms and IoTs, we have analyzed a significantly wider range of IoT based technologies, which can be used in monitoring and diagnosing the COVID-19 symptoms and victims.
c) To conclude, we review the challenges and directions for the future, as well as the need to solve these issues.

### C. Paper Organization

The literature review in this paper is organized as follows: In Section II, we discuss the search strategy used to find the literature on IoT-based COVID-19 diagnosing and monitoring systems. In Section III, background on IoT technology is presented. In section IV, we discuss benefits of IoT technology which can help in slowing down the spread rate of COVID-19 virus. In Section V, several IoTs-based architectures, devices, tools, and technologies are reviewed in details. Section VI presents the lessons learned and future directions. Finally, Section VII concludes our research efforts.

## II. Search Strategy

**FIGURE 1.**
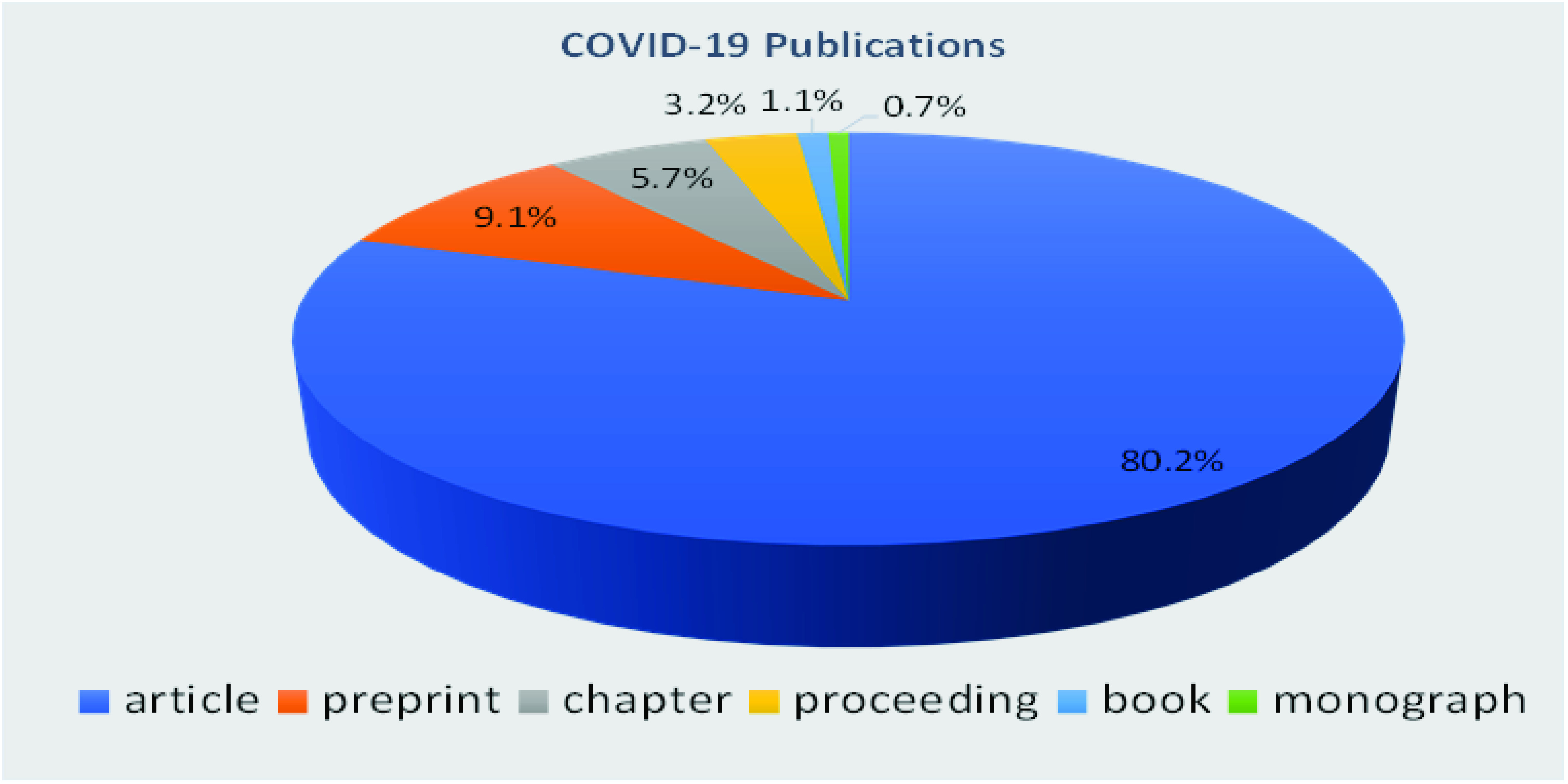
COVID-19 publications worldwide [21].

According to Dimension Database [21], as of 3rd August 2022, scientists from 36,653 organizations have published over 1,206,136 articles about the coronavirus (Fig. 1). Among which, over 40,717 included the phrases of “machine learning algorithms”, “artificial intelligence”, “IoTs”, or “COVID-19 Monitoring” etc, within the title or the abstract. However, we emphasize that the search results may vary and depend on the reference and keyword search.

In this research study, legitimate databases, including IEEE Xplore [39], ScienceDirect [40], ELSEVIER Chaos, Solitons & Fractals [41], SpringerLink [42], ACM [43], and ArXiv [44], NATURE, PUBX, have been utilized to look for COVID-19 papers. Besides, we also utilized Google Scholar [45] search to find the related research articles. The articles are chosen utilizing the keywords IoTs, COVID-19, Coronavirus, deep learning, ML, Artificial Intelligence, COVID-19 forecasting systems, COVID-19 diagnosing systems, and COVID-19 monitoring systems. On 3rd August 2022, we finished the selection of most recent research articles for this survey article.

## III. Background in IoTs Healthcare System

**FIGURE 2.**
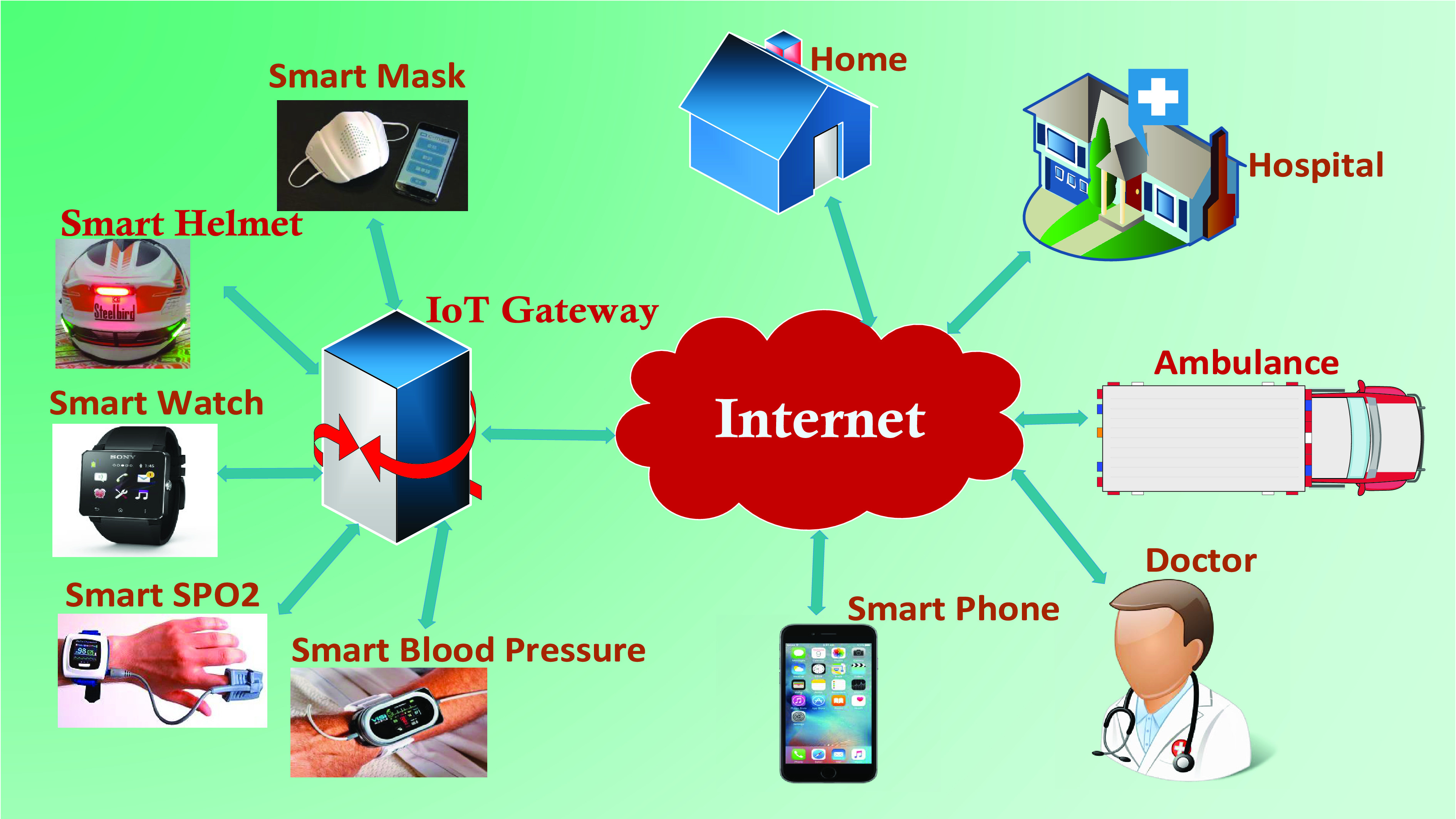
Basic IoT-healthcare architecture.

An IoT is a network of devices that interact with each other through the machine to machine (M2M) correspondences [46], [47], [48], permitting data collection and exchange. The Harvard Business Review article [49], characterizes the IoT devices as “smart, connected devices”. A device is called smart if it is able to gather information from its surroundings, analyze it using ML algorithms, and perform automated actions based on the outcomes without human intervention. These smart devices are connected to an IoT gateway (preferably to a smartphone via Bluetooth) and then the Internet and other technologies for different purposes (Fig. 2). For example, for smart patient tracking [50], [51], [52], to alert an hospital or healthcare authorities when a patient with a heart attack or injury is detected [31], [53], [54].

It can also be used to store detailed health information gathered by wearable sensor nodes such as a smart watch. Then the stored information on the private cloud or edge server can be downloaded by a patient’s physician during a routine exam [55]. A typical smart healthcare IoT system comprises of the following components:

### A. Sensor Nodes

Sensors are the most vital and important components of smart healthcare IoT technology. Sensors collect data and information from surroundings and transfer it over a network to a dedicated central node (server or cloud) for analysis and taking the necessary actions [56]. Sensor nodes are further classified as wearable sensors and vision based sensors:

#### 1) Wearable Sensors

Earlier diagnosis of Corona infection in individuals can stop its widespread across the globe. Wearable sensors can monitor various health and physical fitness related parameters such as heart rate, fever, breathing rate, and blood oxygen level. Therefore, wearable sensors can assist individuals and physicians in predicting whether the patient is infected or not infected. Furthermore, healthcare systems equipped with wearable sensors may assist physicians or surgeons to monitor infected patients or probable, and predict their future symptoms and health risks. With this information, a patient can also assess the severity of the disease and afterwards can contact a hospital or specialist before the health condition gets any worse.

Wearable sensors can assist in monitoring other numerous health conditions including; heart rate and pulse rate [57], blood pressure [58], calorie intake and burnt [59], smart exercises tracking, stress and anxiety tracking [60], menstrual tracking [61], and pregnancy tracking [62], [63], [64]. Commercially several smartwatches, wrist bands, and chest traps are available for tracking an individuals pulse rate, heart rate etc. These include HRM-Tri by Garmin for monitoring heart rate [65], H7 by Polar (heart rate sensor) [66], FitBit PurePulse [67], and TomTom Spark Cardio [68].

#### 2) Vision Based Sensors (Cameras)

They have been installed in physical locations such as around the home, hospitals, isolation wards, and ICUs for continuously monitoring the patients suffering from critical health conditions such as “Parkinson’s Disease” [69]. Similarly, it is revealed by the authors in [70] that progression in “Parkinson’s Disease” can be monitored very efficiently when utilizing wearable sensors in combination with vision based cameras. Besides, the authors recommended that ML algorithms could ultimately prompt improved treatments in the coming future.

The utilization of vision based devices may help in following a patient with Coronavirus and provide care for recovering patients at home. For instance, for diagnosing fever smart vision based systems have been introduced in numerous nations where sensor technology has been integrated with vision based cameras and sends the captured data to the servers or clouds. The framework likewise utilizes Artificial Intelligence to recognize faces and matches them to centralized databases. For instance, the Department of Defense, US has used vision cameras to capture thermal images to identify people with high body temperatures [71].

In China, Baidu, one of the biggest Artificial Intelligence innovators and internet provider companies around the globe, has built computer vision powered infrared cameras to check passengers’ temperatures at Beijing’s Qinghe Railway Station. These cameras can also recognize citizens who are not paying any heed to the preventative measures [72]. These smart camera vision systems are more beneficial than hand-held thermometers because the operators and subjects are not physically near each other and they do not require as much manpower. An equivalent computer vision camera system has been installed in Oxford, England, to screen if people are following the social distancing measures. An AI-based company in the USA called “Landing AI”, which was created by quite possibly the most well known AI specialists on the planet - “Andrew Ng”, has additionally made a vision based social distance detection machine that monitors crowds and alarms the authorities whenever preventative measures are not being followed [38].

### B. IoT Gateway

An IoT Gateway is a physical or virtual device that connects sensors nodes and smart devices to cloud storage. It gives IoT devices access to the Internet. In simple words, it enables communication among smart devices, protocols and technologies. It collects massive data from a number of connected sensor nodes, processes it, and forwards it to the cloud where ML algorithms and AI technology transform it into some meaningful and useful asset. To manage the IoT devices and sensor nodes it also receives data from the cloud. It means all the information going to IoT devices and the cloud or vice versa must go through the connected IoT gateway.

### C. A Smart Fone App

A smart phone app works alongside the client’s mobile phone to gather location information utilizing Bluetooth and to interact with the server through the cellular data network. A smart phone APP is developed to communicate with users. First, the user has to make an account and answer questions pertaining to their background. Then the smart phone app gathers the information through the sensor nodes and sends it to the edge or cloud server for training the data.

## IV. Benefits of IoT Based Covid-19 Monitoring and Diagnosing Systems

An IoT based monitoring and diagnosis system can help in hindering the spread rate of the COVID-19 virus. For instance,

• The wearable sensors can help in monitoring, as well as diagnose the symptoms of COVID-19 disease. These sensors with the assistance of AI techniques can issue a clear warning to the potentially infected individual and the concerned authority to take precautionary measurements, for example, to isolate themselves and to take appropriate tests and avoid social gathering.
• With the assistance of geo-area services and different technological advancements, for example, drone technology and Bluetooth etc., individuals can be cautioned through the alarm on the off chance that they come close to another individual. Additionally, it can help authorities in forcing and maintaining the social distancing in public places through smart sensors and other technologies.
• Governments and health specialists may utilize COVID-19 monitoring systems and information for observing the behavior of individuals after recovering from the corona disease.
• One of the significant advantages of IoTs in health care system is remote monitoring [31]. For instance, SPHERE [31] is a remote monitoring framework that comprises wearable and vision based sensors for monitoring the patients at home. Further, remote monitoring has been utilized effectively in hospitals by doctors, to monitor the old age citizens with non-critical health conditions at home [73], [74]. Likewise, IoTs technology can be very useful to monitor the COVID-19 patients remotely those who are in isolation wards. Moreover, the family of the patient can keep an eye on the COVID-19 patients who are quarantined at home.
• It tends to be valuable in monitoring infected patients at who are quarantined at home or isolated elsewhere. Artificial intelligence assisted sensors can be utilized to help predict whether individuals are contaminated with the infection, in view of symptoms, such as fever, short of breath, and blood oxygen levels.

## V. IoT-Based Emerging Technologies for Monitoring and Diagnosing the Covid-19

**FIGURE 3.**
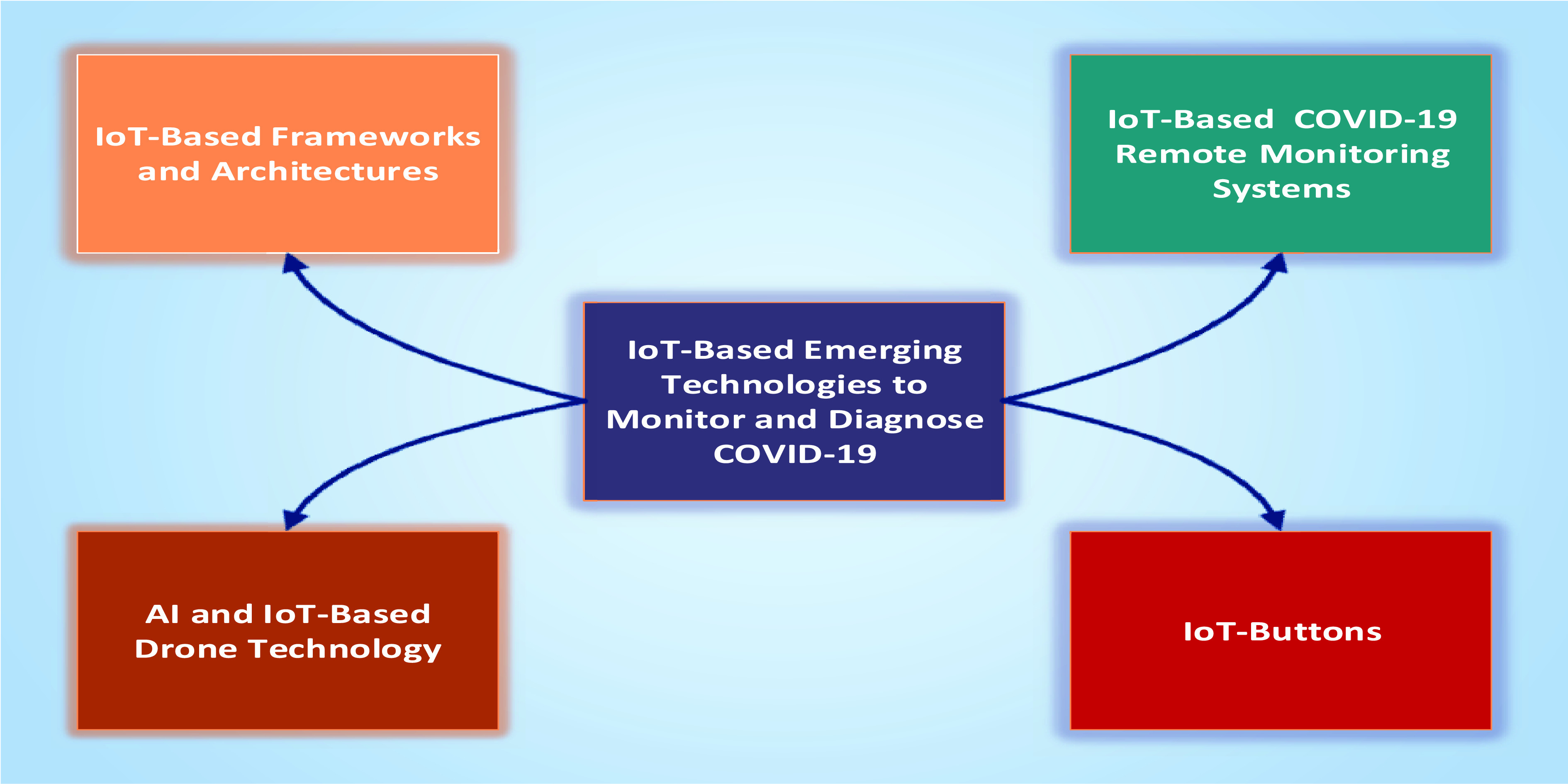
IoT-based emerging technologies for monitoring and diagnosing COVID-19.

To maintain the physical distancing, monitoring health parameters, slowdown the COVID-19 spread, and enforce the precautionary measurements against the COVID-19, different frameworks and architectures, devices, and technologies have been proposed. In this section, we will provide a detail discussion and insights on these technologies (Fig. 3).

### A. IoT-Based Frameworks and Architectures

To observe an individuals’ health condition and notifies them to keep social distance amongst others, the authors in [80] introduced an IoT healthcare system, named “COVIDSAFE”. The proposed framework includes a wearable sensor node that gathers an individual’s health parameters, for example, fever, heart rate, $SpO_{2}$ level, coughing pattern, a smart phone application that acts as a bridge between the user and central server, a Bluetooth assistance based tracking system to alert about the safe distance zone violation, and a voice coughing detector that ceaselessly monitors the individuals voice and records the count of coughs and severity level of the cough. A subgroup of samples from the “Khorshid COVID Cohort (KCC)” [81] study was utilized to create the rules for the decision-making system.

For early COVID-19 detection and to enforce prevention, authors in [82] proposed a multi-layer (6-layer) IoT architecture that connects smart cities, smart hospitals, smart transportation, and smart health care system. Several use cases and algorithms were also proposed and discussed to prevent the COVID-19 spread such as use of smart ventilator and sensor nodes to monitor the oxygen level in confirmed COVID-19 cases, remote COVID-19 patients monitoring in hospitals, isolation wards and homes, smart sanitizing in homes, hospitals, and workplaces to disinfect the virus, smart grocery store for virus free shopping, smart gyms, smart nurseries and child care homes for children’s safety against the COVID-19 disease, to monitor the children’ sleep pattern, breathing, body temperature, and use of AI assisted drones to estimate a six feet distance between individuals using distance measurement sensor nodes.

Authors in [83] propose a multilayer architecture comprising of sensors, actuators, communication system, cloud gateway and big data warehouse. Sensors collect the data from the physical world and transfer it via the Internet to the centralized servers for further analysis and decision making. Actuators enable devices or smart things to produce some response based on the information and data received by the sensor nodes. Communication between the proposed multilayer architecture and the Internet is carried out through a gateway device which is further connected to a cloud gateway. In the big data warehouse, various filtering techniques are applied to the raw data to transform it to some meaningful data. Therefore, data warehouse only caches the structured data. Machine learning is utilized to make decision models of the systems based on requirements and received data.

The authors in [84] proposed a COVID-19 monitoring and detection system that utilized wearable sensor nodes to collect the real time COVID-19 disease symptoms, travel to a highly infected area, and communication with potential carriers of the virus. A dataset of “14251 confirmed COVID-19 cases from the COVID-19 Open Research Dataset (CORD-19) repository [85]” was utilized to train and built the predictive model using eight ML algorithms including, “Support Vector Machine, Neural Network, Naïve Bayes, K-Nearest Neighbor (KNN), Decision Table, Decision Stump, OneR, and ZeroR”. The outcomes demonstrated that each one of the calculations, aside from the “Decision Stumps, OneR, and ZeroR” accomplished an accuracy of over 90%. Utilizing the 5 top algorithms would give accurate and precise recognition of potential positive cases of Coronavirus.

The authors in [86] introduce an IoT-based architecture for contact tracing and illness tracing, which explicitly incorporates symptom-based detection that has been disregarded in past in tracing models. The framework is capable of meaningfully merging real-time symptom information (from IoT sensor nodes) and affirmed Coronavirus cases (from medical tests). This gives a quick and proficient method of detecting the virus, which is ultimately the best solution considering the shortage of resources (e.g Coronavirus test kits). The general framework is made out of four unique stages: T1 is the first stage of the system. Then at T2, each person is checked for: (a). symptoms, such as fever, cough, and fatigue, with their corresponding infection rates, (b). People with the infection or showing high symptoms are recognized. At stage T3, people that have been exposed to other infected patients or people showing high symptoms are recognized depending on their distance. Finally, in stage T4 individuals who have been infected are quarantined, and the exposed people are separated from others.

### B. AI and IoT-Assisted Drone Technology

Drones can and have offered many benefits during the global COVID-19 pandemic. For instance, drones may play a vital role at the time of a quarantine in order to decrease the count of Coronavirus cases by decreasing the correspondence among medical staff with patients and infected areas. They can be very useful in keeping medical staff or non-infected people away from isolated patients. They can also be utilized to get to unreachable or contaminated areas such as hospitals, laboratories, and isolated wards.

Several research efforts in industry and academia have proposed the use of drone technology to monitor, detect, and diagnose the Coronavirus and prevent or slow down its spread rate. Several developed countries including China, India, USA, Australia, and Spain etc., have successfully used drone technology to fight COVID-19 (Table 1).

**TABLE 1:** IoT-Assisted Drone Technology

S#	Name of Company	Country	Budget	Purpose/Benefit
1	Australian Department of Defence and University of South Australia	Australia	$\$ $1.5M	Used drone technology in vulnerable and potentially risky areas such as old age homes and crowded areas, for monitoring and detection of infection and respiratory conditions such as fever, pulse, heart rate, and breathing rate [75] [76].
2	———-	USA	———-	Used Drone technology to deliver medical equipment and medicines to the COVID-19 hotspot zones [77]
3	XAG	China	5-Million- Yuan ( $\$ $715, 000)	Utilized agriculture drones for spritzing disinfectants in order to minimize infection rate [78].
4	DJI	Spain Military	5-Million- Yuan ( $\$ $715, 000)	Utilization of agricultural drones created by “DJI”, a prominent Chinese drone manufacturer, to spray disinfectant over public areas [78].
5	———-	China, New Delhe	———-	Used drone technology to instruct people without masks and enforcing the social distancing [76].
6	———-	China	———-	Used Drone technology with thermal cameras to diagnose the fever in medical staff [76].
7	———-	Spain	———-	Used Drone technology to setup COVID-19 awareness campaigns [79].
8	JD.com	China	———-	Used group of drones to deliver foods in strict lockdown areas [79].
9	Antwork Robotics	China	———-	Used Drone technology for delivering supplies in COVID-19 infected areas in China [77]

For instance, a joint research project [75], worth a budget of up to $\$ $1.5M, by the Australian Department of Defense and University of South Australia is under the development process. The aim of the project is to use drone technology in vulnerable and potentially risky areas such as old age homes and crowded areas, for monitoring and detection of infections and respiratory conditions such as fever, pulse and heart rate, and breathing rate. Similarly, “Antwork Robotics”, is a drone delivery company from China which is owned by Terra Drone, is utilizing drone technology for delivering medical supplies in COVID-19 infected areas in China [76].

The USA is also taking vital steps to utilize drone technology in order to deliver medical equipment and medicines to the COVID-19 hotspot zones [77].

Another drone company “XAG” has also made a “5-million-yuan ( $\$ $715,000)” fund to assist the utilization of their agriculture drones for spritzing disinfectant in order to minimize infection rates. The Spanish military has started implementing the utilization of agricultural drones created by “DJI”, a prominent Chinese drone manufacturer, to spray disinfectant over public areas [78].

As an extraordinary measure against the Coronavirus, drone technology has been used to instruct people without masks to use the mask in China. Likewise, the authorities in New Delhi (India) have used drone technology implemented with a thermal camera and vision camera for monitoring people and crowds [87]. Similarly, drones with thermal cameras are being utilized to monitor fever, which allows medical staff to identify possible infections without having to physically touch individuals who are infected with the virus [76].

Drones can be highly useful for broadcasting vital information in urban areas or regions that don’t have sufficient communication channels. The police authority in Madrid, Spain, utilized a drone that had a speaker fixed to it in order to advise individuals of the guidelines set by the government during the pandemic [79].

The Coronavirus crisis has also allowed drones to transport food. The “Chinese ecommerce company JD.com” has created its group of drones to do several food delivery tests which will replace hour long transportation times with a flight of less than ten minutes [79].

Tracking the geolocation and distance between two or more individuals is another vital and useful feature of drone technology. Therefore, during a pandemic, tracking the distance between individuals can provide valuable data to the government and researchers. This information can be used to determine who the person has been in close contact with. The authors in [88], proposed a multi-layer drone-based Coronavirus monitoring and detection system comprising of thermal imaging system for measuring the social distancing, wearable sensor system for detecting the movement and collection of COVID-19 symptoms, edge computing system for analyzing the data uploaded by the drones and sensor nodes, making the real time decisions, privacy and security system to allow the confidentiality and privacy of an individuals data. Algorithms for multiple use cases including remote monitoring, social distancing, and smart sanitization are proposed and implemented using the real time simulations. In the simulation, the method is tested for indoor and outdoor activities. From the simulation experiments, authors showed that from 3 to 30 drones can cover upto 1200 KM distance in approximately 18,900 minutes.

### C. IOT-Based COVID-19 Remote Monitoring Systems

**FIGURE 4.**
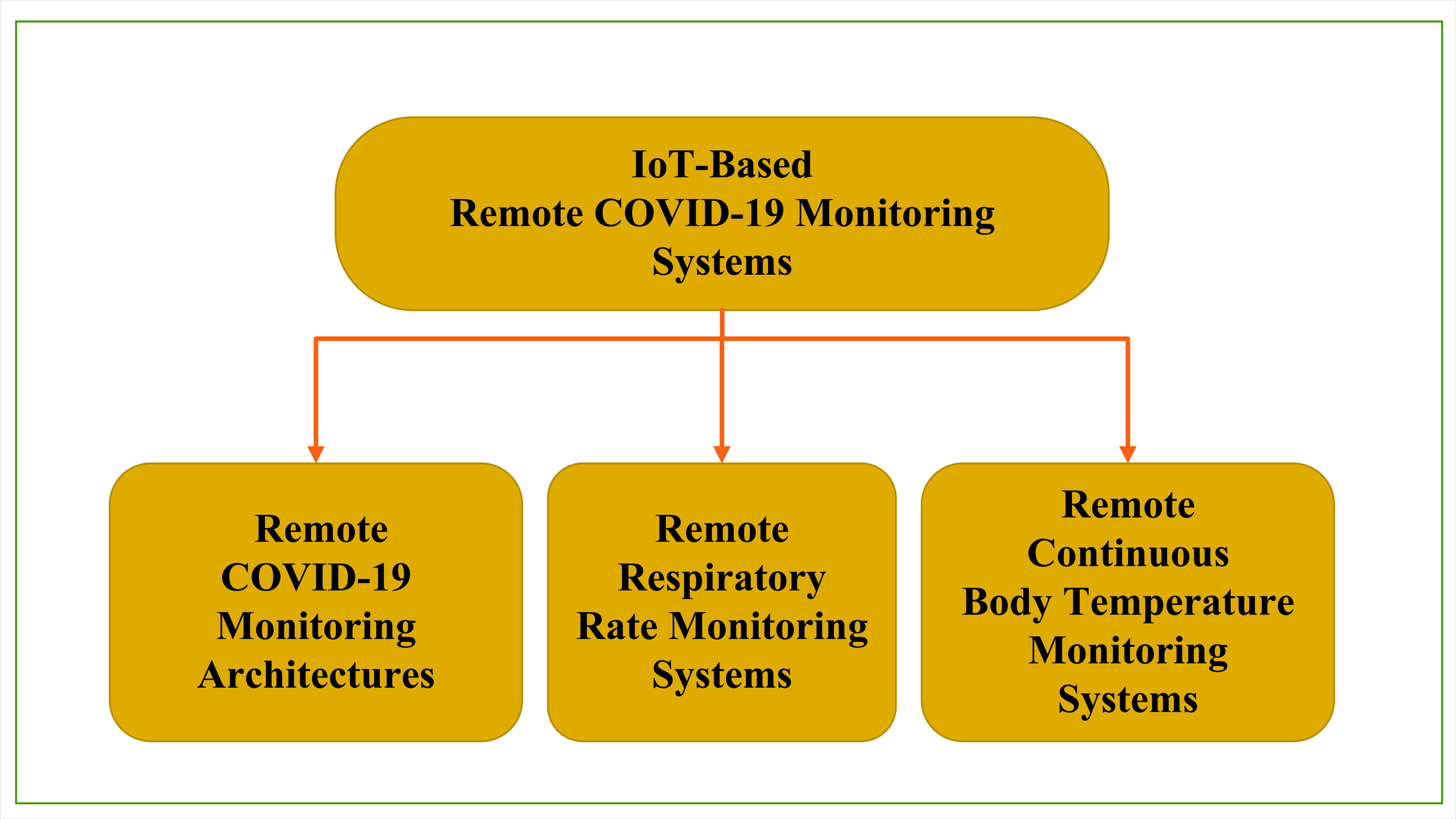
IoT-Based COVID-19 remote monitoring systems.

Remote health monitoring refers to observing a people’s health from outside the clinical settings. During the COVID-19 outbreak, remote monitoring has come in as a innovative and powerful tool for the health officials, government health organizations and public. Remote monitoring can be very beneficial in managing and controlling the COVID-19 pandemic. For instance, remote monitoring system empowers observing individuals from their residence that saves the government expenses and time by measuring the changes in the individual in quarantine for medical readings. It can also be specifically used to collect pandemic data and get real time clinical feedback. Since the start of COVID-19, researchers have initiated remote monitoring architectures, remote respiratory rate monitoring systems, remote continuous body temperature monitoring systems and remote heart rate monitoring systems (Fig. 4). In the following section we provide insights to those systems.

#### 1) COVID-19 Remote Monitoring Architectures

In a pandemic, wearable sensors equipped with AI technologies can be very useful in remote monitoring [58]. For example, if the $SpO_{2}$ level of an individual is less than 85% or the health of the individual is in critical condition, the hospital staff can get alerts through their smart phones or smart alarming systems and then they will be able to send help out to the individual. As a result, patients can get build corrective recommendations pertaining to medicines and other precautionary measures to be followed at their residence through IoT applications.

The authors in [89] propose a remote monitoring architecture that monitors the individuals infected by the Coronavirus and prevents healthcare staff (such as physicians and nurses) from also getting infected. This smart system monitors individuals with the disease based upon putting numerous sensors to take various features of the individual. These parameters include taking the patient’s temperature, respiratory rate, heart rate, blood pressure, and time. It targets two types of individuals: patients with chronic health conditions and individuals with home manageable health conditions such as old age people living in their residence. The authors in [89] propose a framework named “E-Quarantine” that monitors the critically ill patients and forecasts the critical cases in a day by 98.7% based upon the data collected via the sensor nodes such as “blood PH level, heart rate, blood pressure, body temperature, and respiratory rate”.

#### 2) Remote Respiratory Rate Monitoring Systems

According to the world health organization, respiratory rate > 30 breaths/min is a vital symptom for the diagnosis of Coronavirus infection. [90]. At triage level, different values of respiratory rate are used by health officials to make decisions based upon the use of supplemental oxygen. These values are also useful for early recognition of COVID-19 infection and assist in categorizing the patients according to the severity of the infection (mild/moderate/severe). These values are also used as the criterion for the assignment of patients to ICU or isolation wards [91]. COVID-19 Patients require on going vital symptom monitoring, even during daily life. Remote respiratory rate monitoring will help health officials with the timely identification of COVID-19 patients’ deterioration, which leads to the implementation of early intervention methods [92].

There are many existing technological solutions such as sensors for the accurate monitoring of respiratory rate which if combined with the IoTs and AI can be utilized for the remote respiratory rate monitoring of Coronavirus patients. The authors in [93] propose a respiratory rate forecasting system using nasal breath sound recordings with a phone. The proposed method recognizes nasal airflow utilizing a built-in smartphone microphone or a headset microphone that is put beneath the nose. These sound waves or signals can be converted into radio signals and transferred via the high speed 5G technology to edge server or cloud storage for further analysis and making decisions. Patients that need real time symptom monitoring, even during daily life, may be provided with wearable sensor technology such as smart garments. Unlike most of the mentioned technologies, smart garments may give precise respiratory rate values even during everyday life [94]. Likewise, The authors in [95] studied the performance of a multi-sensor smart clothing technology during routine workouts (walking and running).

#### 3) Remote Continuous Body Temperature Monitoring Systems

Numerous analysts have effectively proposed wearable sensor technology for persistent fever monitoring which is also an important symptom used for the diagnosis of COVID-19 patients. These smart body temperature monitoring devices can take advantage of emerging technologies and can be utilized for isolated monitoring of infected patients. For instance, based on several artificial neural networks, the authors in [96] proposed a wearable system which continuously checks the body temperature with very high accuracy and efficiency. The authors in [97] presented a contactless continuous body temperature monitoring method in which they utilize a single thermal camera and deep-learning based face detection techniques to detect the forehead temperature of the individuals. The trial results show that the general mean absolute error (MAE) and root-mean squared error (RMSE) of the proposed structure contrasted and mechanical instruments are 0.375 °C and 0.439 °C, respectively.

The authors in [98] developed a small and comfortable wearable sensor device to continuously monitor the body temperature of a baby. The body temperature readings are transferred to the parents through a wireless communication system for the purpose of remote monitoring via the mobile phone. Another IoT based gadget known as Health Companion utilizing wearable computing was introduced in [99] which monitors the temperature and heartbeat on a regular basis. This gadget intends to gather various boundaries of the human body, helps individuals to monitor their health, and works with specialists to examine the patients’ symptoms. The sensor warns the individual as well as the clinical staff if there is a significant increase in temperature or the individual has a fever.

### D. IoT Buttons

IoT button is another technology that can be use in the crisis of COVID-19. IoT buttons are small, integrated, and Wi-Fi enabled devices that when pressed can send timely notifications or alerts to some central body for taking further actions or activate some pre-specified tasks.

A well-known example of IoT button is Amazon’s “Dash” buttons that was firstly designed and developed for the amazon customers to supply the depleted products at their location such as, to re-ordering the detergent when it is running low at the laundry [100]. AWS IoT (Amazon Web Services Internet of Things) is Amazon’s new reprogrammable and Wi-Fi enabled IoT button that one can program to control the internet-connected devices and services [101]. According to the Amazon’s announcement new AWS button can be utilized as a remote control for TVs, a check-in/check-out device for customers at hotel, easily order food, to get into a car, open doors, call a taxi, police, or people, track chores around the house, order prescriptions or items, or control home apparatuses as though individuals were utilizing a controller. For instance, if the $SpO_{2}$ level of the patient is under 85%, clinic experts will get alarms through information driven applications and afterward send additional consideration administrations to the patient. Patients can likewise get fabricate restorative suggestions in regards to medications and additional precautionary measures to be carried out at their own residence through IoT applications. He/she can likewise add his/her relatives or companions to share his/her PGHD with various degrees of access control because of safety and protection concerns.

By and large, because of security concerns, a patient probably won’t consent to share his/her information, and in such cases, wellbeing gadgets will send information on an entryway gadget (e.g., patient’s telephone). Nonetheless, a few cautions can be bogus positives, and to address such blunders, thick sensor networks alongside clinical gadgets can assist with lessening bogus positives. Besides, characterization methods like Hidden Markov Model (HMM) can be utilized to group abnormalities in PGHD. This model can be sent on cloud or door to distinguish bogus cautions.

In hospitals or homes, IoT buttons can be configured to carry out a wide range of actions. For instance, in the event that the medical issue of an affirmed COVID-19 case detached at home or in ICU deteriorates, by squeezing the catch, a medical services supplier will be cautioned, or relatives will be advised if there should be an occurrence of a crisis. IoT buttons can be used to keep up with high cleaning principles and cutoff the quantity of emergency clinic gained contaminations (HAIs) [102]. They can be use to issue alerts to management when hand sanitizers deployed at hospital facility need refilling, sanitation certain area, maintenance issues, cautioning them of any disinfection or other various issues that might present a danger to public security.

## VI. Challenges and Future Directions

Based on the benefits and suitability of IoTs in healthcare industry, we have investigated a few exploration endeavors towards creating IoT based COVID-19 monitoring and diagnosis health care systems. We have discussed numerous research efforts towards developing IoT-based COVID-19 remote monitoring systems along the wearable sensor nodes, IoT buttons, drone technology, and many architectures and frameworks proposed to control and manage the COVID-19 outbreak.

In terms of sensor nodes, we found that, a lot of suitable options are available to monitor various COVID-19 symptoms such as body temperature, respiration rate, heart rate, temperature, and oxygen saturation level. We also found that, the healthcare systems equipped with wearable sensors and vision-based sensors may assist physicians and individuals to monitor infected patients or probable, and predict their future symptoms, assess health risks, and forecast their future conditions. However, development of integrated sensor technology for diagnosing and predicting COVID-19 disease need substantial attention from the research community in terms of accuracy, availability, wearability, and cost. There are still no all-in-one sensor devices available that can monitor and predict all COVID-19 symptoms. A considerable attention of researchers from academia and industry is required to further enhance the quality of sensor nodes in terms of cost, reliability, wearability, and accuracy.

Research interests need to be focus on developing respiration rate, oxygen saturation, continuous body temperature monitoring devices which are more wearable than placing them on chest walls (smart patches), under the nose or on fingers and which also produce hospital-grade accuracy equivalent results without compromising energy efficiency and wearability and deployable worldwide. Likewise, we also concluded that continuous body temperature monitoring devices are extremely useful diagnostic tools and should be used and promoted in the current pandemic. However, their accuracy is limited by the degree of contact with the human body. More they are contacted with skin, more accurate results are achievable. However such devices are uncomfortable for patients. The authors in [103] and [104] proposed to embed the temperature sensing devices in textile to achieve the higher level of accuracy and comfortability. Therefore, engineers can also focus on developing smart textiles for measuring body temperature until some alternative or electronic printed patches on some soft backing support is manufactured. Furthermore, despite the numerous advantages of wearable devices, ML algorithms have not been explored in this area and providing a significant research opportunities.

In terms of remote monitoring systems, we found that it can assume a significant part with regards to the COVID-19 pandemic. It might work with medical services help for holed up COVID-19 patients just as for other individuals that have limited access to clinical benefits. The improvement of remote monitoring would likewise support medical care administrations and consequently will reduce the burden from the healthcare facilities. However, successful implementation of remote monitoring system would require support of high speed cellular networks to upload/download the health related data with ultra low latency to or from sensors to an external storage (such as cloud or edge server) [105], [106]. In this regard, use of 5G technology can allow to develop responsive and dynamic remote monitoring system. 5G is the fifth generation of wireless communication technology and expected to provide better performance in terms of higher speed, lower latency, wider range, increased availability, and more reliability [107], [108], [109]. The integration of remote healthcare monitoring system with advanced 5G technology has the potential to revolutionize the healthcare industry and also provide significant research opportunities to computer scientists, data scientist, and telecommunication industry.

## VII. Conclusion

Today, millions of people have been infected and dieing around the globe due to deadly Coronavirus. Currently, Corona vaccines from many developed countries are under trail and are being utilized in more than 199 countries. Around 70% of world’s population needs to be vaccinated in order to reach herd immunity. Although life saving corona vaccination campaigns are in full swing around the world, yet it would require countless efforts from the science community and government organizations to hinder its spread around the globe. This is due to the fact that new variants are emerging day by day. There are currently five notable variants: alpha, beta, gamma, delta, and Mue. As new variants emerge, immunity provided by vaccines and boosters is threatened. Therefore, besides vaccines, other methods and technologies need to be utilized in order to overcome this pandemic. There have been massive efforts in various research areas and a wide variety of tools, technologies and techniques have been explored and developed to combat the war against this pandemic. Interestingly, IoTs technology was one of the first areas to be used to combat Covid-19. Up till now, several real-time and intelligent IoT-based COVID-19 diagnosing, and monitoring systems have been proposed to tackle the COVID-19 pandemic. To assist the research community by imparting an overall comprehension of the continuous exploration and potential research areas in COVID-19, in this survey article, we provide a comprehensive review of IoTs technology to develop COVID-19 diagnosing, and monitoring systems. Based on our in-depth literature review, we analyzed a number of IoT based technologies which can be used in diagnosing and monitoring the infected individuals. Furthermore, we identified the challenges and also provided our vision about the future research on COVID-19.
